# Ratsnake: A Versatile Image Annotation Tool with Application to Computer-Aided Diagnosis

**DOI:** 10.1155/2014/286856

**Published:** 2014-01-27

**Authors:** D. K. Iakovidis, T. Goudas, C. Smailis, I. Maglogiannis

**Affiliations:** ^1^Department of Informatics and Computer Technology, Technological Educational Institute of Lamia, 35100 Lamia, Greece; ^2^Department of Digital Systems, University of Piraeus, 18534 Piraeus, Greece

## Abstract

Image segmentation and annotation are key components of image-based medical computer-aided diagnosis (CAD) systems. In this paper we present Ratsnake, a publicly available generic image annotation tool providing annotation efficiency, semantic awareness, versatility, and extensibility, features that can be exploited to transform it into an effective CAD system. In order to demonstrate this unique capability, we present its novel application for the evaluation and quantification of salient objects and structures of interest in kidney biopsy images. Accurate annotation identifying and quantifying such structures in microscopy images can provide an estimation of pathogenesis in obstructive nephropathy, which is a rather common disease with severe implication in children and infants. However a tool for detecting and quantifying the disease is not yet available. A machine learning-based approach, which utilizes prior domain knowledge and textural image features, is considered for the generation of an image force field customizing the presented tool for automatic evaluation of kidney biopsy images. The experimental evaluation of the proposed application of Ratsnake demonstrates its efficiency and effectiveness and promises its wide applicability across a variety of medical imaging domains.

## 1. Introduction

Image-based computer-aided diagnosis (CAD) systems aim to aid medical diagnosis by evaluating medical images as objectively as possible, utilizing image features and prior knowledge about the respective application domain. Such systems typically integrate image *segmentation* methods to isolate regions of interest (ROIs) corresponding to salient objects, and automatic *annotation* methods, to assign labels that characterize each region. Prior knowledge is usually obtained from related medical studies and multiple domain experts, through manual segmentation and annotation of images of that domain. Contemporary data annotation systems are based on *semantic web* technologies and take advantage of knowledge representation structures, called *ontologies*, that enable formal, unambiguous semantic annotation, which can also be used for knowledge inference [1]. According to this approach, labeling involves semantic, instead of plain textual, object identifiers. In what follows, for readability purposes, the manual image segmentation and annotation processes will be referred to as *graphic image annotation*.

Graphic image annotation is usually a time consuming process because it requires interaction of the domain expert with the corresponding annotation software tool, whereas the required effort can be thought as a function of the aimed annotation detail and the annotator's skill. In [2] we presented Rapid image annotation with snakes (Ratsnake) as an open access, cross platform software tool (Ratsnake is available at http://innovation.teilam.gr/ratsnake/), implementing a framework for efficient graphic annotation of multiple images of the same context that contributes to the reduction of both the annotation time and cost. The efficiency of this tool relies on a simple graphical user interface (GUI), featuring complementary graphic annotation protocols and a properly modified snake model [3], which in its original form enables semiautomatic image segmentation. The customizability of the snake model makes Ratsnake versatile and applicable to a variety of imaging domains. Image annotation is complemented by semantics, formally represented in ontologies that can either be developed for a particular application or retrieved from the semantic web. The functionality of Ratsnake has been later extended to automatic annotation of multiple segmented images by integrating an ontology of qualitative spatial semantics and a reasoning engine for inference of the annotations [4, 5].

**Figure 1:**
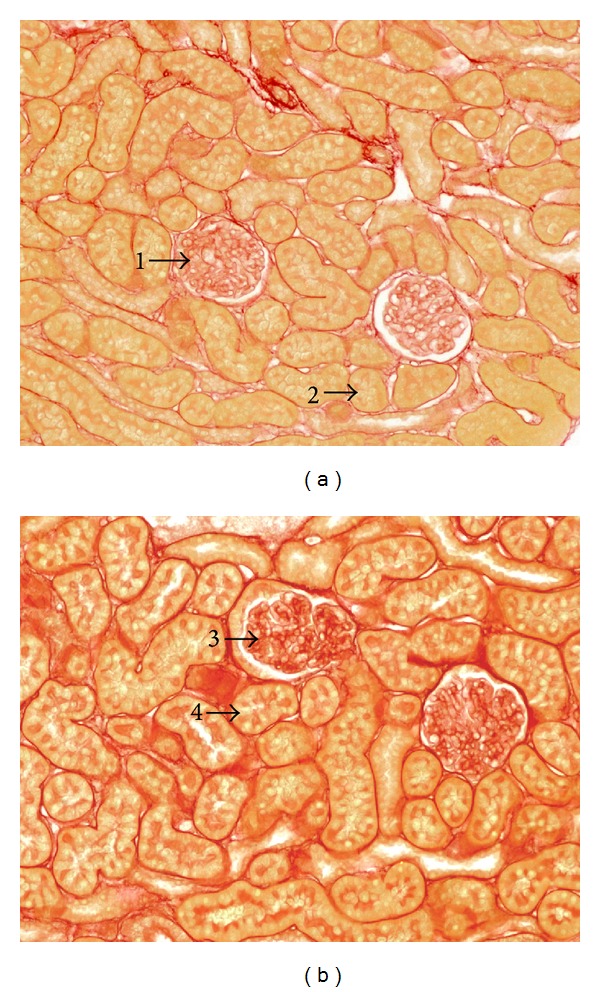
Salient objects in kidney biopsy images. The arrows indicate the regions of interest. (a) Normal biopsy: (1) nonpathogenic glomerulus; (2) nonpathogenic tubulus. (b) Pathogenic biopsy: (3) pathogenic glomerulus; (4) pathogenic tubulus.

In this work we focus on a methodology that can turn Ratsnake into a fully functional CAD system. The comparative advantage of this approach is that it enables faster development of such systems as plugin modules that can exploit Ratsnake's segmentation, semantic annotation, ontological inference, and measurement capabilities that have been introduced in its latest version. To this end we present a novel application and case study, which can also be considered as a model for developing future CAD systems based on Ratsnake. The CAD system presented in this paper aims at fast evaluation of microscopy images from kidney biopsies. These images are very complex, in the sense that, unlike other types of medical images, their content is characterized by diverse, inhomogeneous regions, densely, not a priori distributed over the image space (Figure 1). A machine learning algorithm has been incorporated to include prior knowledge about the imaging domain of kidney biopsies within the customizable snake model and generate an image force field evaluating textural image features. This force field can be considered as a saliency map derived from the classified image samples, roughly indicating boundaries of ROIs, which guides the snake model to finely segment and automatically annotate these ROIs.

The rest of this paper consists of five sections.  Section 2 provides background information about the medical application considered.  Section 3 reviews the previous works related to our study. The proposed graphic image annotation framework and the methodology considered for its customization for kidney biopsy image analysis are described in Section 4. The results from the experimental evaluation of Ratsnake are apposed in Section 5 and the conclusions that can be derived are summarized in the last section.

## 2. Medical Background

Kidney biopsy images can provide an estimation of pathogenesis in the obstructive nephropathy disease [6]. Obstructive nephropathy is the main cause of renal failure, which occurs in all ages but is often met in children and infants. It is caused by obstruction of the urinary tract, with hydronephrosis (which is dilation of the renal pelvis and calyces resulting from obstruction of flow of urine), slowing the glomerular filtration rate and tubular abnormalities. Considering that obstructive nephropathy is not a rare disease [7], computer-aided evaluation of the pathogenic areas on a kidney biopsy image is very useful for the proper assessment of the disease. In this context, the modified Ratsnake tool is able to accurately annotate salient objects and regions of interest in the examined images, such as the most important kidney structures, namely, glomerulus and tubulus. The goal is to classify regions as pathogenic or not (see Figure 1). The quantification of obstructive nephropathy can be achieved by detecting and measuring the alterations that occur in these prominent kidney structures. In addition, the size of these objects is an important feature that can be measured utilizing the proposed tool. Glomeruli have a diameter ranging from 50 to 120 *μ*m [8], while the tubules of the nephrons are 30–55 mm long [9] with an average diameter of 50 *μ*m. The dilatation of the glomerulus and tubulus objects is a symptom of obstructive nephropathy [6], which can be measured using the modified Ratsnake tool. Furthermore, the tool can be used to monitor the disease progress or the effects of drugs and other therapeutic procedures by comparing and measuring images from follow-up studies. Accurate and mainly repeatable quantification of obstructive nephropathy by an expert is a rather difficult task, and since it is not a rare disease with severe implication in children and infants, a tool able to provide fast and reproducible results, like the one presented in this work, is considered valuable. It should be noted that such a tool for detecting and quantifying the disease is not yet available to our knowledge.

## 3. Related Work

A variety of software tools have been proposed to aid medical diagnosis and support medical decision making [10]. Image-based CAD tools rely on image segmentation and annotation methods, which are applied either in an automatic or a semiautomatic framework. The majority of these tools are application-specific; for example, DoctorEye [11] is an annotation tool proposed for fast semiautomatic annotation of tumors in magnetic resonance imaging (MRI), and Arthemis [12] has been proposed especially for the annotation of colonoscopy images.

State-of-the-art CAD systems and methods for microscopy include a real-time decision support system for diagnosis of rare cancers [13]; a system for discrimination of normal from benign thyroid nodules in cytological images [14]; a system for detection and grading of carcinoma in histology images [15]; a method for prostate cancer diagnosis and grading [16]; a web-based software framework for segmentation of cervical cell nuclei in high-resolution microscopy images [17]; and a tool for classification of biological microscopic images of lung tissue sections with idiopathic pulmonary fibrosis [18]. These works indicate that texture plays an important role in the characterization of the content of microscopy images and that machine learning can be effective for automatic annotation of such images. Additional works could be referenced, but to the best of our knowledge there is no other work in the literature related to kidney biopsy evaluation, except the preliminary works of our research group [19, 20], which have been performed on a limited dataset, addressing automatic characterization of objects in obstructive nephropathy images.

In this study we accept the challenge to exploit Ratsnake, which is a generic, extensible image annotation tool, to develop a novel CAD expert system for the evaluation of kidney biopsy images. The approach we follow can also be considered as a paradigm for the development of similar applications across a variety of medical imaging domains. Generic image annotation tools relevant to Ratsnake [2] include LabelMe [21], Photostuff [22], Photocopain [23], K-space Annotation Tool (KAT) [24], ImageParsing.com [25], Graphic Annotation Tool (GAT) [26], Caliph [27], and M-Ontomat Annotizer [28].

LabelMe is a web-based image annotation tool with a very usable GUI. The main limitations of the online version of LabelMe include inability to annotate images without publicizing them and slow response times if the user's internet connection is slow. These problems can be overcome by setting up LabelMe on a local server; however, this is a quite complex procedure for the average user. The semantic annotations of LabelMe are based on free text or a lexical database called WordNet [29]. Photostuff has a more complex GUI that enables ontology-based semantic annotation of images, in web ontology language (OWL). Photocopain is intended mainly for semantic image annotation in resource description framework schema (RDFS) language or OWL since the graphic tools it provides are only of fixed shape (rectangle or oval). KAT is a rather flexible annotation tool enabling not only high but also low level semantic image annotation using the Core Ontology of Multimedia (COMM) [30], and it features a framework for semiautomatic labeling of image regions by classification. ImageParsing.com is a commercial solution to graphic image annotation. It provides semiautomatic image segmentation capabilities accessible through a much more complex GUI than that of the other image annotation tools, whereas semantics are not considered in the annotation process. ImageParsing.com is a commercial solution to image annotation based on ImageParser and VideoParser annotation tools which feature semiautomatic image segmentation functions (hierarchical image parsing), but they are not publicly available. Annotations are provided by specialized personnel only through a paid web service, and only a fraction of annotated datasets are provided freely through its website. Semantic web standards to formalize data extracted from images are not supported. GAT is a publicly available annotation tool that combines both semiautomatic image segmentation and semantically aware annotation, and it can also be used for annotation of multiple images or image sequences. It uses partition trees to navigate through image segments, which are automatically defined at different spatial scales by a hierarchical region merging approach. Caliph is an image annotation tool suitable for the creation of new MPEG-7 image metadata. The MPEG-7 description supported by Caliph consists of the following parts: metadata description, creation information, media information, textual annotation, semantics, and visual descriptors. However, since MPEG-7 is an XML format, Caliph is not compatible with formal semantic formats and services, such as OWL. The capabilities of M-Ontomat Annotizer are compatible with those of GAT but it utilizes a much simpler “Magic Wand” method [26] for semiautomatic segmentation of approximately uniform image regions. A concise review study of other annotation tools can be found in [31].

A summary of the described state-of-the-art generic annotation tools is provided in Table 1. Ratsnake displays several advantages over the state-of-the-art image annotation tools, which can be summarized to the following: (a) it enables rapid graphic annotation of ROIs using a grid-based freehand approach, usually requiring only a single mouse drag by the user [2]; it features a customizable, easily extensible, snake-based framework for semiautomatic image segmentation; (c) it provides the ability to semantically annotate arbitrary-shaped ROIs using any OWL ontology available in the semantic-web, to automatically construct ontologies of spatial relations between annotated objects; (d) it uses these ontologies to automatically infer annotations of unknown objects in image sequences of a static context (e.g., the different organs projected in X-rays are characterized by static spatial relations) [4]; (e) its latest version enables area measurements and comparisons between annotated regions for evaluation of graphic annotations. All these advantages have made Ratsnake the annotation tool of choice for fast implementation of image-based CAD systems. The methodology proposed in the following section, which is integrated in Ratsnake as a plugin, enables Ratsnake to automatically annotate kidney biopsy images of nonstatic context.

## 4. Methodology

**Figure 2:**
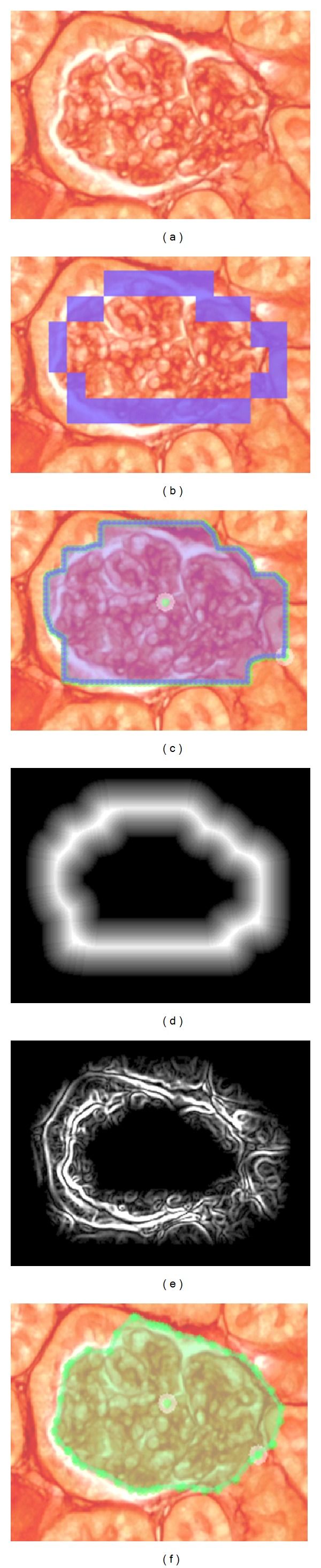
Example of segmentation and annotation of the pathogenic kidney biopsy image illustrated in Figure 1(b) using Ratsnake. (a) Pathogenic glomerulus region of Figure 1(b). (b) Quick rough freehand initial user annotation. (c) Polygon user annotation with landmarks automatically derived from the freehand annotation. (d) *T*(*B*_*d*_). (e) *P*_*I*_^*cs*^ defined by (2). (f) Segmented ROI using (2) with *f*_1_(*I*) and image-specific snake parameters. However, such an image-specific approach would not be suitable for a CAD system capable of coping with annotation of any images of this kind.

The segmentation framework of Ratsnake considers that the user initially provides a quick, rough, outline of a ROI (Figure 2(a)) which is subsequently refined by a parametric *active contour model*, also referred to as snake [32] (Figure 2(b)). This snake-based framework is now enhanced by the introduction of a force field generated by a machine learning-based method. This force field is implemented as a Ratsnake plugin and attracts the deformable contour towards the boundaries of a target classified ROI. The details of this approach are provided in the rest of this section.

### 4.1. Generic Snake-Based Image Segmentation Framework

A snake is a time-varying parametric curve of the form *v*(*s*, *t*) = (*x*(*s*,*t*), *y*(*s*,*t*))^*T*^ where *x* and *y* represent coordinate functions of *s* ∈ [0, *L*] and time *t* in the image plane. Given an image *I* with a size of *N* × *M* pixels with values in *Ω*⊆*R*, the energy functional that dictates the shape of the snake is given by *E*(*v*) = *S*(*v*) + *P*(*v*), where

(1) $$\begin{array}{c} S\left(v\right)=\frac{1}{2}\overset{L}{\underset{0}{\int }}\left(\alpha {\left|\frac{\partial v}{\partial s}\right|}^{2}+\beta {\left|\frac{\partial^{2}v}{\partial s^{2}}\right|}^{2}\right)ds,\\ P\left(v\right)=\overset{L}{\underset{0}{\int }}P_{I}\left(v\right)ds \end{array}$$

represent the *internal* and the *external energy* forcing the contour to move. In (1), *α* and *β* are weight parameters controlling the continuity (or tension) and the curvature (or rigidity) of the contour, respectively. Typically, the snake algorithm considers a scalar function for the generation of the external *force field* estimated as *P*_*I*_(*v*) = −*γ* · ||∇*I*|| or *P*_*I*_(*v*) = −*γ* · ||∇(*G*_*σ*_∗*I*)||. In these equations *γ* is a weight parameter, *G*_*σ*_ is a 2D Gaussian function, and *σ* is its standard deviation. The user may guide the evolution of the snake by adding constraining terms to *P*_*I*_(*v*). Many recent snake models are based on this snake model but use different force fields leading to improved segmentation results. Representative examples include the gradient vector field [33] and the boundary vector field models [34], which efficiently cope with the well-known limitations of the original snake model [3]. Such limitations include the capture range and the extraction of concave objects.

Considering these limitations and the fact that different applications have different requirements (e.g., with respect to the target objects, their boundaries, and their backgrounds), Ratsnake incorporates a customizable function for force field generation. In its general form this function is defined as

(2) $$\begin{array}{c} P_{I}^{cs}\left(v\right)=\left(1-e^{\eta \cdot T(B_{d}(v_{0}))}\right)\cdot \Pi_{f}\left(v\right), \end{array}$$

where

(3) $$\begin{array}{c} \Pi_{f}\left(v\right)=-\overset{\Delta }{\underset{i=1}{\sum }}\delta_{i}\cdot f_{i}\left(I\right) \end{array}$$

and *f*_*i*_ : *Ω* → *Ω* is a user-defined preprocessing function of *I*, such that the force driving the snake towards the boundaries of the target object increases, and *δ*_*i*_ is a weight parameter that controls the degree to which *f*_*i*_(*I*) contributes to the external energy. For *f*_1_(*I*) = −*γ* · ||∇(*G*_*σ*_∗*I*)||, the force field of the original active contour is obtained. *T*(*B*_*d*_) denotes the Euclidean distance transform (EDT) applied on image *B*_*d*_ obtained by erosion of the binary image produced by the projection of the (interpolated) contour *v*_0_ = *v*(*s*, 0) on the image plane for all *s* ∈ [0, *L*]. This transform is introduced to attract the contour towards the users' graphic annotation, assuming that they intuitively try to approximate the boundaries of the target object. This formulation is generic and can be used for the implementation of the force field *P*_*I*_(*v*) of the original snake model or of a more recent snake model such as BVF [34] or even of a future model of this kind. Functions *f*_*i*_ can be easily implemented as plugin modules of Ratsnake in simple Java. The minimization of *E*(*v*) is solved by the greedy algorithm proposed in [35], which is computationally efficient.

Figure 2 illustrates an example segmentation of a pathogenic Glomerulus region using Ratsnake according to (1) and (2), with snake model parameters tuned specifically for that particular image. However, this set of parameters is unlikely to be suitable for the segmentation of other regions of this kind due to the complexity of the kidney biopsy images and would not be acceptable in the context of a CAD system for everyday practice. To cope with kidney biopsy segmentation more robustly, that is, using a common set of snake parameters for most of the images of this kind, we introduce a new force field term *f*_2_(*I*) in (2), generated as described in the next paragraph.

### 4.2. Force Field Generation for Segmentation and Annotation of Kidney Biopsy Images

In kidney microscopy images the pathology is located mostly within salient anatomy objects (i.e., glomerulus and tubulus) so in this context the first step is the recognition of such objects in the examined image dataset. Since the edges that separate the targeted regions are not very clear, the proposed methodology based on active contours and adaptable force field generation is considered appropriate for handling this task. The adaptable force field term *f*_2_(*I*) in (2) is generated by supervised pattern classification. The classification model is developed using prior knowledge about kidney biopsy images and their ROIs (Figure 1), obtained from a set of training images manually annotated by domain experts. The patterns for model training and classification of new, not previously annotated regions of the images to be evaluated by Ratsnake are generated as described below.

#### 4.2.1. Image Representation

Color information is discarded by 8-bit grey-scale conversion, considering that the luminosity of kidney biopsy images explains a significantly larger variance (more informative) than color image components. As it can be noticed from the indicative images of Figure 1 the image hues are rather constant, with a very small variance only in the red scale.

The images are raster-scanned and square blocks (subimages), smaller than the ROIs, are uniformly sampled. From each sampled block a set of textural features, forming a feature vector, are extracted for image representation. First- and second-order statistical measures were considered as image features [36]. By following the best first-feature selection strategy [37] we selected the following subset of features as the most informative for the particular application: the mean and the standard deviation of the block intensities, the contrast, the inverse difference moment, the correlation, the entropy, and the angular second moment.

#### 4.2.2. Machine Learning-Based Image Annotation

Prior knowledge about the medical imaging domain of interest from the experts is introduced by machine learning. To this end a maximum margin kernel classification approach has been adopted, considering their generality and robustness in the sense that their performance is not easily affected by sparse or noisy data and that they resist to overfitting and to the “curse of dimensionality” [38]. According to this approach learning is based on a quadratic programming optimization procedure which aims at the identification of a subset of important feature vectors from the training set, used for the construction of a separating hypersurface between the two classes. In summary this algorithm proceeds as follows.

Let *I* be an input space of vectors *x*_*i*_, *i* = 1,2,…, *n*, distributed to two classes, labelled as *y*_*i*_ ∈ {−1, 1}. Considering Φ as a nonlinear mapping from the input space *I*⊆*ℜ* ^*ν*^ to an Euclidean space *H*, the training results in finding a hypersurface are defined by the equation

(4) $$\begin{array}{c} w\Phi \left(x\right)+w_{0\;}=0 \end{array}$$

so that the margin of separation between the two classes is maximized. The maximum margin hypersurface is obtained for

(5) $$\begin{array}{c} w=\overset{n}{\underset{i=1}{\sum }}\lambda_{i}y_{i}\Phi^{T}\left(x_{i}\right) \end{array}$$

and *w*_0_ is estimated from the Karush-Kuhn-Tucker complementarity condition. The variables *λ*_*i*_ are Lagrange multipliers which are estimated by maximizing the Lagrangian

(6) $$\begin{array}{c} L_{D}=\overset{n}{\underset{i=1}{\sum }}\lambda_{i}-\frac{1}{2}\overset{n}{\underset{i=1}{\sum }}\overset{n}{\underset{j=1}{\sum }}\lambda_{i}\lambda_{j}y_{i}y_{j}K\left(x_{i},x_{j}\right) \end{array}$$

with respect to *λ*_*i*_. The vectors *x*_*i*_, for which 0 < *λ*_*i*_ ≤ *c*, are selected for the construction of the separating hypersurface. Parameter *c* is a positive constant. As *c* increases a higher penalty for errors is assigned. Function *K*(*x*_*i*_, *x*_*j*_) is known as kernel function; it is defined as the inner product

(7) $$\begin{array}{c} K\left(x_{i},x_{j}\right)=\Phi^{T}\left(x_{i}\right)\Phi \left(x_{j}\right) \end{array}$$

that should satisfy Mercer's condition [38].

Most commonly used kernel functions are the linear *K*(*x*_*i*_, *x*_*j*_) = *x*_*i*_ · *x*_*j*_, the polynomial *K*(*x*_*i*_, *x*_*j*_) = (*γ*·*x*_*i*_·*x*_*j*_+1)^*p*^ of second (*p* = 2) and third order (*p* = 3), and the Radial Basis Functions (RBF) *K*(*x*_*i*_, *x*_*j*_) = *e* ^−||*x*_*i*_−*x*_*j*_||^2^/*γ*^, where *γ* is a strictly positive constant. The linear kernel is less complex than the polynomial and the RBF kernels. The RBF kernel enables high-dimensional data sets to be approximated by Gaussian-like distributions similar to those used by RBF networks. The hypersurface separating the two classes is derived by the following equation:

(8) $$\begin{array}{c} \overset{}{\underset{\forall i:0<\lambda_{i}\leq c}{\sum }}\lambda_{i}y_{i}K\left(x_{i},x\right)+w_{0}=0. \end{array}$$

Then, given a test vector *x*, the trained classifier outputs a label *Y*:

(9) $$\begin{array}{c} Y=\mathrm{sign}\left(\overset{}{\underset{\forall i:0<\lambda_{i}\leq c}{\sum }}\lambda_{i}y_{i}K\left(x_{i},x\right)+w_{0}\right), \end{array}$$

which designates the class that *x* belongs to.

**Figure 3:**
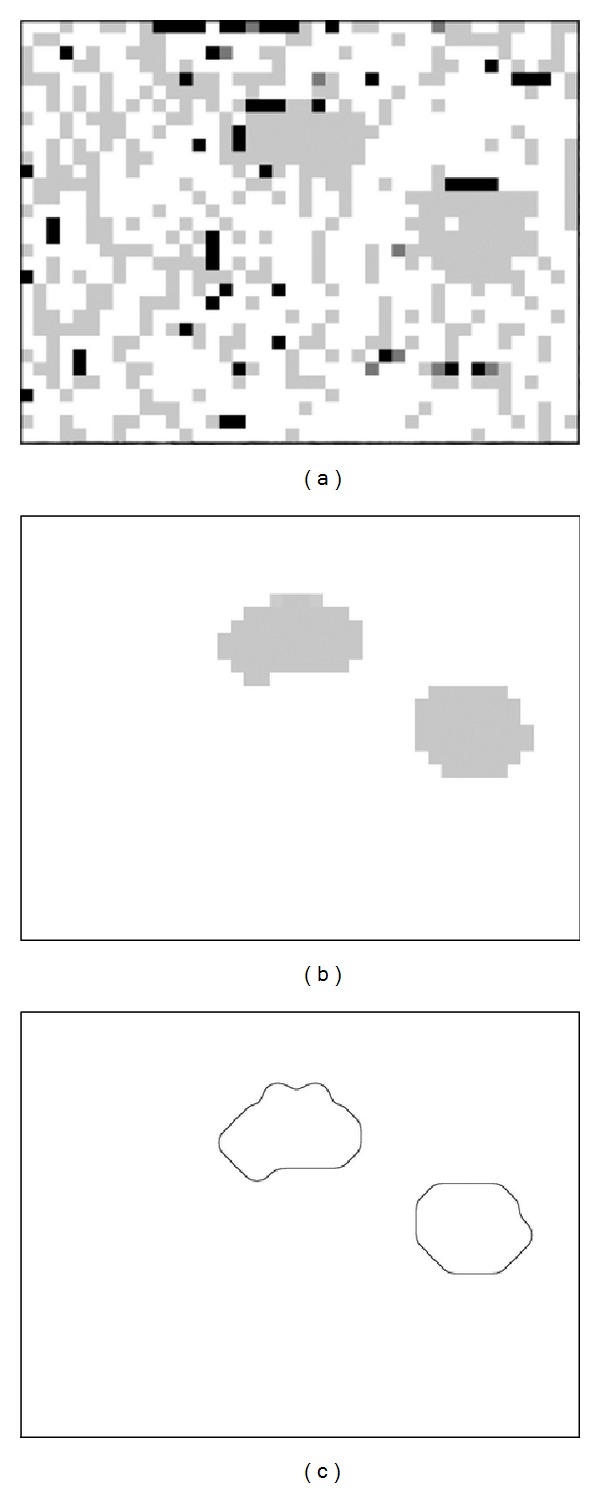
Force field generation for the segmentation of the kidney biopsy image illustrated in Figure 1(b). (a) Classifier's output image, where the different greylevels used indicate different class memberships. (b) Classifier's output after postprocessing with the majority-voting algorithm. (c) Generated force field term *f*_2_(*I*) after postprocessing (the image has also been inverted for presentation purposes).

The kernel classifier trained with representative samples from the training images assigns to each block of the images under evaluation a class label. The annotated blocks of such an image are then represented using different greylevels that indicate their class membership, thus rendering an output image as the one illustrated in Figure 3(a). It can be noticed that several misclassified regions may exist that could be considered as noise artifacts.

#### 4.2.3. Postprocessing

In order to remove noise from the output image, a majority-voting scheme with a dynamic vote limit [39] is applied. The result of this postprocessing operation is illustrated in Figure 3(b). It can be noticed that the edges of the image regions corresponding to the salient foreground objects are quite rough, due to the block-based classification approach used. Therefore, this segmentation result is not suitable itself for accurate measurement of the ROIs.

The force field that will guide the snake towards the actual boundaries of the foreground objects is generated from the resulting image, by three additional postprocessing operations: (a) Gaussian filtering for smoothing of the object boundaries, (b) adaptive thresholding for image binarization, and (c) Canny edge detection [40]. The result of these operations is illustrated in Figure 3(c).

## 5. Experiments and Results

**Figure 4:**
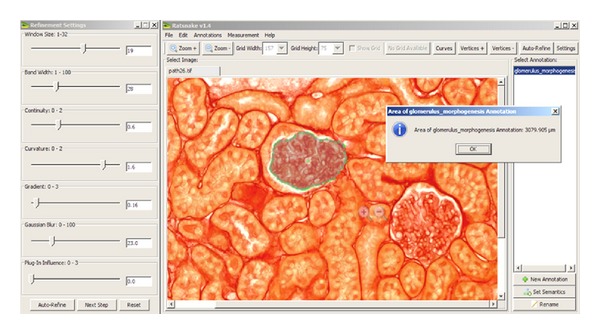
A snapshot of Ratsnake's GUI. It displays the annotated kidney biopsy image of Figure 1(b). The user may click on the annotation names (labels) on the right and display the respective ROI. The labels used are semantic identifiers from the gene ontology [41]. Only the currently selected annotation can be displayed at a time, as a layer over the image to which it belongs. The dialog box is the result of the menu option Measurement→Area Measurement, which is used to measure the area of the current ROI. The panel on the left controls the parameters of the snake.

The described methodology has been implemented in Java and has been integrated in Ratsnake as a custom coded plugin. The processes related to the kidney biopsy image analysis have been implemented as a web service communicating with the plugin. A snapshot of Ratsnake's GUI is illustrated in Figure 4. The effect of the force field generated by the plugin, as well as the values of the rest of the parameters of the snake (see (1)), is controlled by the settings panel. The functionality of Ratsnake from the user's viewpoint for the particular application can be summarized into the following steps.

1. Training Ratsnake for the first time:
  - domain experts use Ratsnake, without any prior domain knowledge loaded to the system, to produce ground truth annotations on a set of representative images selected to be used for training;
  - during graphic annotation of each training image the users may choose to combine manual annotation with the autorefinement option that executes the snake algorithm so as to obtain faster, closer estimates of the target object boundaries; the process of manual and automatic refinement may be repeated until the actual boundary of the object is correctly approximated;
  - the training images along with their annotations are saved in a system's folder.
2. Using the trained Ratsnake for evaluation of kidney biopsy images:
  - either experts, or not-so-experienced domain specialists, who may not be able to safely characterize the objects in kidney biopsy images, can use Ratsnake to quickly select (usually with only a single quick mouse drag) a ROI that roughly includes the object they would like to evaluate.
  - the users utilize the autorefinement option with the effect of the plugin set to a nonzero value; this activates the use of prior domain knowledge collected from the training images; then Ratsnake automatically segments the target object and assigns it a label with its characterization; training is performed only once for a given training set. If this set is changed, then the classifier is retrained with the updated training set;
  - The users may choose the measurement options of Ratsnake to (a) calibrate the system to the preferred measurement units; (b) measure the area of the annotated objects; (c) compare the annotated areas.

Extensive experiments were conducted to demonstrate the effectiveness of the proposed methodology incorporated in Ratsnake for the evaluation of the kidney microscopy images and the achieved annotation efficiency. The dataset considered in this study consists of 60 images, half of which originate from pathogenic kidney biopsies and the rest from healthy (control) kidney biopsies. All images are accompanied with ground truth annotations performed manually by three experts using Ratsnake (with the plugin implementing the proposed method being disabled). The annotations were performed on a conventional laptop with Intel Core 2 Duo 1.83 Ghz 2 MB L2 cache processor and 3 GB RAM. The biopsy samples were stained with the Sirius Red technique, which is one of the most common techniques of collagen histochemistry. In bright-field microscopy collagen is red to pale yellow, while nuclei are ideally black but may often be grey or brown. In the examined kidney images the pathological findings are connected with alterations in the imaging of the two major salient objects, tubulus and glomerulus, which are the major parts of the kidney for the processing of its renal function. The images were acquired with a Nikon Eclipse E400 microscope with Nikon lens Plan Fluor 20x/0.50; Differential Interference Contrast (DIC) microscopy M; ∝/0.17 Working Distance 2.1; and a Microfire by Optronics camera with the following settings: exposure 10 ms; red: 105; green: 100; blue: 100; gain: 1; luminosity: 50; contrast: 60.

The capability of the proposed supervised Ratsnake approach to evaluate kidney biopsy images is assessed by measuring its performance in the classification (automatic annotation) and segmentation of ROIs. All images were raster-scanned and 37 × 37 pixel block samples were obtained. This block size has been selected heuristically, as the most appropriate for providing satisfying accuracy and acceptable processing times, based on pilot experimentation.

The classification of the blocks was based on the kernel classifier described in Section 4.2.2, and its performance was compared with three other widely known classifiers, namely, Naïve Bayes [42], K-Nearest Neighbor [43], and Decision Trees [44]. Tenfold cross-validation has been adopted as a widely accepted method to assess classification accuracy [45]; that is, the dataset was randomly split into 10 mutually exclusive subsets, leaving out one set for testing and using the other nine as training, exhaustively, until all of them serve as testing sets. The best performing classifier for the current problem is the less complex kernel classifier, with linear kernel, achieving a 94.7% accuracy using cost parameter *c* = 1.5, after grid search of the parameter space. The results obtained per class are presented in Table 2, where class precision and recall refer to the capability of the classifier to identify relevant image samples and to correctly label them, respectively [46].

Table 2 indicates that the classification methodology incorporated in Ratsnake enables automatic annotation of ROIs very accurately, despite the type of the objects of interest considered, and performs best for the annotation of nonpathogenic tubulus objects.

In order to assess the segmentation performance of the proposed supervised Ratsnake approach we consider the Jaccard index, which expresses the *overlap ω* between the areas *a*_1_ and *a*_2_ of two shapes (in pixel units), as defined by the ratio

(10) $$\begin{array}{c} \omega =\frac{\alpha_{1}\cap \alpha_{2}}{\alpha_{1}\cup \alpha_{2}} \end{array}$$

which is a standard, well-grounded measure of segmentation accuracy [47]. The settings used from Ratsnake's settings panel (Figure 4) include *d* = 8, *a* = 1.44, *β* = 1.58, *δ*_1_ = 0.6, *σ* = 23, and *δ*_2_ = 0.99. Ratsnake received the above settings after repeating the experiments several times and storing the best performing settings. In each run, several different initial contours have been tested approximately indicating the region to be segmented and annotated.

**Figure 5:**
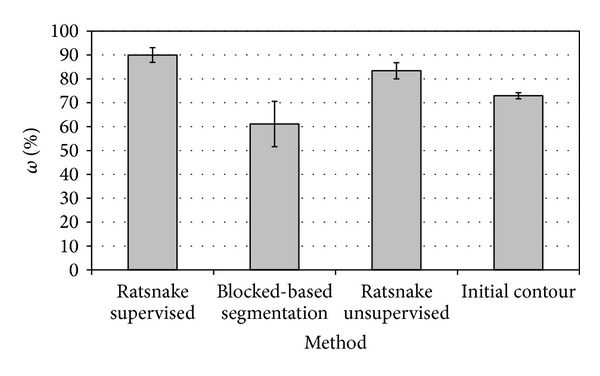
Bar-chart graphically illustrating the results presented in Table 3.

The average segmentation performance of the supervised Ratsnake approach was measured on each object of the available test images, in comparison with the segmentation performance of the unsupervised Ratsnake, that is, with the plugin being disabled, using only *f*_1_(*I*), and the segmentation performance of the block-based classification approach used for the generation of the force field. The results obtained are apposed in Table 3 and graphically illustrated in Figure 5. In the last row of this table, the average overlap of the initial contours manually drawn by the (nonexpert) users to indicate the respective ROI is also provided. It can be noticed that the best performing method is the supervised Ratsnake approach. The block-based segmentation results are low, indicating that the error introduced by the use of image blocks is significantly high; therefore, the results validate that this approach is inadequate for area measurements. Despite its low accuracy it provides an effective force field for the supervision of Ratsnake. As compared with the initial contour, the overlaps obtained by both the supervised and the unsupervised Ratsnake approach indicate a significant contribution of the snake algorithm.

**Figure 6:**
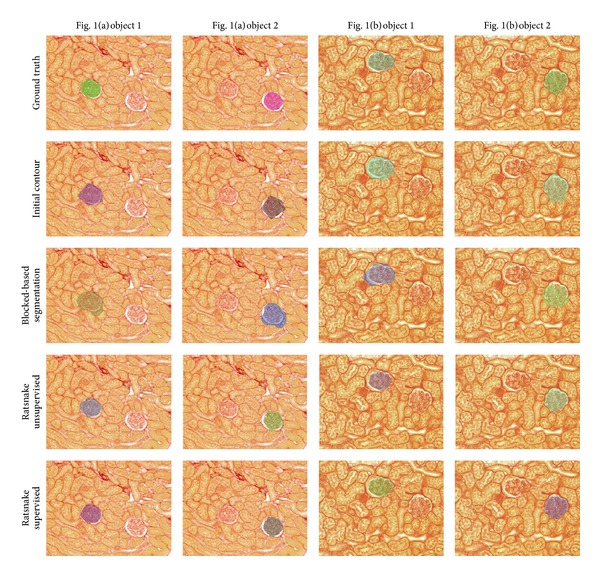
Representative segmentation results obtained using different methods for objects in the kidney biopsy images of Figure 1.

Figure 6 illustrates representative segmentation results obtained for the images of Figure 1, validating the average results obtained. The respective quantitative results in terms of overlap are apposed in Table 4.

The capability of Ratsnake to incorporate prior knowledge about the imaging domain under investigation provides an additional advantage over the state-of-the-art image annotation tools, in terms of annotation efficiency, that is, the time required by the user to annotate the ROIs. In order to demonstrate this advantage we have asked three domain specialists to annotate the dataset using both Ratsnake and LabelMe [17] (running on a local server). The average annotation time required per image using the supervised Ratsnake was 14.5 ± 0.8 seconds, whereas, for the manual LabelMe approach, for the same level of segmentation accuracy, this time reached 21.4 ± 2.53 seconds. However, it should be noted that the time measurements for LabelMe have taken into account only the graphic image annotation times and not the time required by the specialists to decide about the class membership of the graphically annotated ROIs (which is automatically performed by the supervised Ratsnake based on its prior domain knowledge). This time cannot be sufficiently estimated in the scope of this study since it may include literature searches or even interaction between specialists, for example, for a second opinion, which is undoubtedly a time consuming process. Therefore, the use of Ratsnake can contribute in faster evaluation of the kidney biopsy images.

## 6. Discussion and Conclusions

In this paper, we presented a novel approach to the development of image-based CAD systems. This approach exploits Ratsnake, a generic, versatile, and open access image annotation tool, for fast development of such systems as plugin modules. A Ratsnake plugin module should implement only the part of the expert system required for the description of prior knowledge about the application domain of interest. In order to demonstrate this unique capability we presented a novel medical application with impact on the diagnosis and quantification of obstructive nephropathy, through computer-aided evaluation of kidney biopsy images. This is considered a nontrivial task, which is not fully supported by a specialized computer-based annotation tool such as the one presented in this paper. The proposed methodology is based on a machine learning approach to include prior knowledge about kidney biopsy images, so that the user of Ratsnake can be able to quickly segment a ROI, estimate its actual boundaries, measure its area, and automatically annotate it with a semantic identifier corresponding to a diagnostic characterization, that is, pathogenic or not. The results showed that the utilization of machine learning to supervise Ratsnake has a significant impact on the segmentation accuracy of kindey biopsy images, enabling it to perform more accurate area measurements efficiently. The evaluation of a kidney biopsy image based on a classification model obtained by training is quite efficient as it involves only linear complexity algorithms.

As an annotation tool, Ratsnake can be used for efficient generation of ground truth training data (graphic annotations), which are directly accessible by the CAD system and can be used to actively update its domain knowledge for improved diagnostic performance. Considering its capability to embed ontologies [2], the annotations produced by Ratsnake can be associated with semantic identifiers described in biomedical ontologies [48], enabling unambiguous representation and semantic interoperability with relevant medical systems, such as clinical information systems. Given a set of graphically annotated images and a semantically annotated training image, the semantic identifiers of the graphically annotated images can be automatically inferred [4, 5], speeding up the generation of a semantically annotated training data set.

Currently the image segmentation process is based on the original snake model, but the fact that the force field of Ratsnake is customizable enables the implementation of other recent or even future snake models for more accurate segmentation. The customizability of Ratsnake makes it suitable for a multitude of applications involving image segmentation, annotation, and image-based measurements, across a variety of imaging domains. Future work includes further automation of the image annotation process by smart snake initialization and implementation of an extensible web service featuring active learning capabilities that will be able to provide Ratsnake with knowledge on various imaging domains.

## Figures and Tables

**Table 1:** Comparative summary of state of the art generic image annotation tools.

Image annotation tool	Graphic annotation	Semiautomatic segmentation	Image sequences	Semantic annotation	Annotation method	Measurements	Free
Ratsnake [2, 4]	Polygon Grid/freehand	Customizable snake model	Yes	Free text OWL	Manual Automatic	Yes	Yes
LabelMe [21]	Polygon	None	No	Free text WordNet	Manual	No	Yes
Photostuff [22]	Fixed shape Polygon	None	No	OWL	Manual	No	Yes
Photocopain [23]	Fixed shape	None	No	RDFSOWL	Manual	No	Yes
KAT [24]	Fixed shape Polygon	None	No	RDFS OWL	Manual	No	Yes
ImageParsing.com [25]	Polygon	Hierarchical image parsing	Yes	Free text WordNet	Manual Automatic	No	No
GAT [26]	Fixed shape Polygon	Partition trees	Yes	MPEG-7 OWL	Manual	No	Yes
Caliph [27]	Polygon	None	No	Free text	Manual	No	Yes
M-OntoMat Annotizer [28]	Fixed shape Polygon	Magic Wand	Yes	RDFS DAML	Manual	No	Yes

**Table 2:** Confusion matrix obtained by the linear kernel maximum margin classifier.

	True nonpathogenic glomerulus	True nonpathogenic tubulus	True pathogenic glomerulus	True pathogenic tubulus	Class precision (%)	Total accuracy (%)
Pred. nonpathogenic glomerulus	381	1	12	12	**93.8**	
Pred. nonpathogenic tubulus	4	244	0	0	**98.4**	
Pred. pathogenic glomerulus	8	0	312	12	**94.0**	
Pred. pathogenic tubulus	4	3	14	314	**93.7**	
Class recall (%)	**96.0**	**98.4**	**92.3**	**92.9**		**94.7**

**Table 3:** Average **ω** measured with respect to the ground truth using different image segmentation methods.

Method	Average *ω* (%)
Ratsnake supervised	90.0 ± 3.1
Block-based segmentation	61.1 ± 9.5
Ratsnake unsupervised	83.4 ± 3.4
Initial contour	72.9 ± 1.3

**Table 4:** **ω** values obtained for the images from Figure 6.

Image			Ratsnake supervised	Block-based Segmentation	Ratsnake unsupervised	Initial contour
Figure 1(a)	**ω** (%)	Object 1	92.8	59.3	86.8	71.3
		Object 2	91.0	56.3	81.4	73.5
Figure 1(b)	**ω** (%)	Object 1	89.1	70.8	84.5	71.7
		Object 2	92.9	76.6	86.4	72.6
